# Osteoprotegerin (OPG), The Endogenous Inhibitor of Receptor Activator of NF-κB Ligand (RANKL), is Dysregulated in *BRCA* Mutation Carriers

**DOI:** 10.1016/j.ebiom.2015.08.037

**Published:** 2015-09-09

**Authors:** Martin Widschwendter, Matthew Burnell, Lindsay Fraser, Adam N. Rosenthal, Sue Philpott, Daniel Reisel, Louis Dubeau, Mark Cline, Yang Pan, Ping-Cheng Yi, D. Gareth Evans, Ian J. Jacobs, Usha Menon, Charles E. Wood, William C. Dougall

**Affiliations:** a Department of Women's Cancer, UCL EGA Institute for Women's Health, University College London, 74 Huntley Street, London WC1E 6AU, UK; b Department of Pathology, Keck School of Medicine, USC/Norris Comprehensive Cancer Center, University of Southern California, Los Angeles, CA, USA; c Department of Pathology, Section on Comparative Medicine, Wake Forest School of Medicine, Winston-Salem, NC 27157, USA; d Department of Molecular Sciences, Amgen Inc, Seattle, WA 98119, USA; e Department of Genomic Medicine, Institute of Human Development, University of Manchester, St. Mary's Hospital, Manchester M13 9WL, UK; f The University of New South Wales, UNSW, Sydney, NSW 2052, Australia; g Therapeutic Innovation Unit (TIU), Amgen Inc., Seattle, WA 98119, USA

**Keywords:** Breast cancer, *BRCA1* and *BRCA2* mutations, RANKL, OPG, Carcinogenesis, Cancer prevention

## Abstract

Breast cancer development in *BRCA1*/*2* mutation carriers is a net consequence of cell-autonomous and cell nonautonomous factors which may serve as excellent targets for cancer prevention. In light of our previous data we sought to investigate the consequences of the *BRCA*-mutation carrier state on RANKL/osteoprotegerin (OPG) signalling.

We analysed serum levels of RANKL, OPG, RANKL/OPG complex, oestradiol (E2), and progesterone (P) during menstrual cycle progression in 391 *BRCA1*/*2*-mutation carriers and 782 noncarriers. These studies were complemented by analyses of RANKL and OPG in the serum and mammary tissues of female cynomolgus macaques (n = 88) and serum RANKL and OPG in postmenopausal women (n = 150).

*BRCA*-mutation carriers had lower mean values of free serum OPG in particular in *BRCA1*-mutation carriers (p = 0.018) compared with controls. Among *BRCA1*/*2* mutation carriers, lower OPG levels were associated with germline mutation locations known to confer an increased breast cancer risk (p = 0.003). P is associated with low OPG levels in serum and tissue, particularly in *BRCA*-mutation carriers (*rho* = − 0.216; p = 0.002). Serum OPG levels were inversely correlated (*rho* = − 0.545, p < 0.001) with mammary epithelial proliferation measured by Ki67 expression and increased (p = 0.01) in postmenopause.

The P–RANKL/OPG system is dysregulated in *BRCA*-mutation carriers. These and previously published data provide a strong rationale for further investigation of antiprogestogens or an anti-RANKL antibody such as denosumab for breast cancer prevention.

## Highlights

• Osteoprotegerin (OPG) is the endogenous inhibitor of Receptor Activator of NF-κB Ligand (RANKL)
• RANKL has been shown to be crucially important in progesterone-mediated breast carcinogenesis
• Serum OPG is regulated by progesterone and low in *BRCA1*/*2* mutation carriers
• Low serum OPG is associated with increased proliferation in the mammary gland
• Antiprogestogens or anti-RANKL antibody (denosumab) may be new strategies for breast cancer prevention in *BRCA1*/*2* carriers.

Preventing deadly cancers is a high priority of 21st century medicine. To find the most promising cancer causing targets which can be modulated using chemo-preventive drugs remains the biggest challenge. In this paper we show that OPG, which is the physiological antagonist of RANKL (a factor known to be crucially involved in breast carcinogenesis), is low in women who have inherited a gene mutation (i.e. in *BRCA1* or *2*) which puts them at extremely high risk of developing breast cancer. An antibody which mimics OPG might be a very attractive option in preventing breast cancer.

## 1 Introduction

Risk factors for breast cancer development include genetic predisposition and exposure to elevated sex steroid hormones. Germline mutations in *BRCA1*/*2* account for elevated sex steroid hormones and 2%–10% of breast cancer cases depending on ethnic origin (Fackenthal and Olopade, 2007). Hormonal risk factors include early menarche, late menopause or first full pregnancy, weight gain, and combined hormone replacement therapy (HRT) (Veronesi et al., 2005). Evidence suggests a potential relationship between *BRCA* mutations, sex hormone levels, and end-organ effects to hormones, and cancer risk (Kim and Oktay, 2013, Segev et al., 2015, Titus et al., 2013, Widschwendter et al., 2013). High breast cancer risk in *BRCA*-mutation carriers is particularly evident premenopausally (relative risk 32 and 10 for 40–49-year-old *BRCA1*/*2*-mutation carriers, respectively) (Robson and Offit, 2007), whilst removal of both ovaries in premenopausal *BRCA1*/*2*-mutation carriers markedly reduces breast cancer risk (Domchek et al., 2010). Studies in *BRCA1*/*p53*-deficient mice indicate a direct role for progesterone (P) in mediating mammary tumourigenesis (Poole et al., 2006). Specific signalling pathways mediating interactions between *BRCA*-associated risk and sex steroid exposure have not been identified.

The relationship between higher serum sex steroid levels and increased breast cancer risk is well established in postmenopausal women (Fourkala et al., 2012, Key et al., 2011), but documenting a premenopausal link is challenging due to hormonal fluctuations associated with menstrual cycle progression (Key et al., 2013, Kaaks et al., 2014). The temporal dynamics of oestradiol (E2), P, and other hormones during the menstrual cycle may be more important for breast cancer risk than absolute hormonal levels at a single time point (Brisken, 2013). Moreover, hormonal context, life stage, and reproductive status may significantly impact hormone-associated risk: transient exposure to high levels of P in women with multiple pregnancies has not been associated with increased breast cancer risk, whereas persistent exposure to relatively low progestin levels postmenopausally has been associated with breast cancer in epidemiologic (Beral, 2003) and clinical (Chlebowski et al., 2003) studies.

*BRCA* mutations may lead to cell-autonomous defects including defects in chromosome duplication and cytokinesis (Venkitaraman, 2014). Although cell nonautonomous alterations such as hormonal alterations leading to aberrant growth of hormone-sensitive target cells may be particularly relevant to sporadic cancers (Veronesi et al., 2005), recent evidence linking *BRCA*-associated risk and hormonal factors (Domchek et al., 2010, Dubeau, 2015) suggests an interplay between cell-autonomous and cell nonautonomous factors in *BRCA*-mutation carriers. Preclinical studies (Chodankar et al., 2005, Hong et al., 2010, Yen et al., 2012) show mice carrying a *BRCA1* mutation in the steroid-hormone-producing granulosa cells have a longer pro-oestrous phase, corresponding with the oestrogen-dominant follicular phase of the human menstrual cycle, as well as elevated basal E2 levels and evidence of increased oestrogen exposure in target organs such as bones. Recently, we demonstrated altered endometrial thickness and higher E2 and P levels in well-defined parts of the luteal phase in *BRCA*-mutation carriers vs wild-type controls (Widschwendter et al., 2013). Targeting cell nonautonomous hormonal targets may thus be an effective, noninvasive strategy for breast cancer prevention in *BRCA*-mutation carriers. A majority of breast cancers among *BRCA1*-mutation carriers are oestrogen-receptor negative (Foulkes et al., 2004), and removal of ovaries in *BRCA1*-mutation carriers with an oestrogen-receptor negative breast cancer dramatically reduces breast cancer-specific mortality (hazard ratio 0.07, p = 0.009) (Metcalfe et al., 2015), suggesting that breast cancer biology in *BRCA1*-mutation carriers is determined by ovarian hormones other than oestrogens. Hence, strategies targeting cell nonautonomous oestrogen pathways (i.e. selective oestrogen receptor modulators like tamoxifen or aromatase inhibitors), although successful for primary prevention and adjuvant treatment in oestrogen receptor-positive cancers (Cuzick et al., 2015, Cuzick et al., 2014, Forbes et al., 2008), are unlikely to be successful in *BRCA1*-mutation carriers. Therefore, we aimed to explore alternative pathways as targets for breast cancer prevention in *BRCA* carriers.

Receptor activator of nuclear factor kappa-B ligand (RANKL), a member of the tumour necrosis factor (TNF) superfamily, plays a key role in bone remodelling and immune function. RANKL is an important mediator of sex hormone-driven mammary gland development, proliferation, and carcinogenesis (Gonzalez-Suarez et al., 2010, Schramek et al., 2010, Wood et al., 2013). Blocking RANKL (Gonzalez-Suarez et al., 2010, Schramek et al., 2010, Joshi et al., 2010) or progesterone-receptor (PgR) pathways (Poole et al., 2006) substantially reduces mammary cancers in mice. In bone, one of the other main sources of RANKL and its physiological antagonist osteoprotegerin (OPG), evidence suggests a direct and inverse tissue/serum relation for OPG and RANKL (Findlay et al., 2008).

Here we analysed the dynamics of serum ovarian hormones, free RANKL, OPG, and the RANKL/OPG complex in *BRCA*-mutation carriers and wild-type controls throughout the menstrual cycle to test whether RANKL and OPG are dysregulated in *BRCA*-mutation carriers. We further examined whether hormonal exposure modulates serum RANKL and OPG not only in serum, but also in mammary tissues, and the relationship between RANKL/OPG and mammary epithelial proliferation in cynomolgus monkeys.

## 2 Materials & Methods

### 2.1 Subjects and Design

#### 2.1.1 Human

Subjects were participants in the UK Familial Ovarian Cancer Screening Study (UKFOCSS), recruited from 42 regional centres from June 2002–September 2010 after ethical approval (Eastern MREC 97/5/007). Included subjects were > 35 years old with known *BRCA* status (Widschwendter et al., 2013), provided serum samples, no previous/subsequent history of cancer or intrauterine device, not used oral contraceptives during the collection period, and provided the dates of their last menstrual period. We enrolled 391 *BRCA1*/*2*-mutation carriers meeting these criteria, and 782 noncarrier volunteers matched for age and day of menstrual cycle. All samples were stored in liquid nitrogen and tested at the same time. Additionally, we analysed serum from 50 and 150 healthy premenopausal and postmenopausal women (obtained from Cureline, S. San Francisco, CA), for which premenopausal criteria was defined as age < 45 years or ≥ 3 consecutive normal menses immediately before blood collection; postmenopausal criteria was defined as age > 52 years or last menses ≥ 12 months before blood collection.

All women gave consent for their samples to be used for research.

#### 2.1.2 Animal

Archived samples were used from an experiment (Cline et al., 2002) involving 6–8-year-old, ovariectomised, adult female cynomolgus macaques (*Macaca fascicularis*) randomised to diets containing placebo (control; n = 31), 0.042 mg/kg conjugated equine oestrogens (CEE; n = 28), or CEE + 0.167 mg/kg medroxyprogesterone acetate (CEE + MPA; n = 29) over 2 years. Dose equivalents approximated standard HRT doses of CEE (0.625 mg/day) and MPA (2.5 mg/day). Serum and mammary tissue samples were collected at study end. Immunohistochemistry was performed as previously published for Ki67 and PgR (Cline et al., 2002) or RANK/RANKL antigens (Wood et al., 2013). H-scores, obtained by multiplying staining intensity (scales 0–3) with the number of positive cells (range, 0–300), were documented for epithelial tissues from each group for each antibody (Cline et al., 2002). Mammary gland expression of *Ki67* (*MKI67*), *RANKL* (*TNFSF11*), and *OPG* (*TNFRSF11B*) mRNA was evaluated using real-time quantitative PCR, as previously described (Wood et al., 2013). All procedures involving macaques in the original study were conducted per state and federal laws and standards of the US Department of Health and Human Services and approved by the Wake Forest University Animal Care and Use Committee.

## 3 Procedures

### 3.1 Serum Steroid Hormone Assays

Serum E2 and P from randomly mixed and masked batches of mutation carriers and controls were measured with automated immunoassays on an Elecsys 2010 analyser (Roche Diagnostics GmbH, Mannheim, Germany) (Widschwendter et al., 2013). E2 and P intra-assay coefficients of variability (CV) were 1.6%–5.7% and 1.5%–2.7%, while interassay CVs were 2.3%–6.2% and 3.7%–5.4%, respectively.

### 3.2 RANKL, OPG, and RANKL/OPG Serum Assays

Free soluble RANKL (sRANKL), free soluble OPG (sOPG), and sRANKL/OPG complex were measured by ELISA. The assays were validated for dilutional linearity, sample parallelism, accuracy, and precision. sRANKL capture was achieved with recombinant human OPG (R&D Systems, Minneapolis, MN, Cat# 185-OS/CF) precoated onto a microplate, while sOPG capture was achieved by precoating microplates with soluble recombinant huRANKL (R&D Systems, Cat# 390-TN/CF). Postwashing, RANKL and OPG were detected by incubations with biotinylated monoclonal antibodies (Amgen Clones 80A9 & 78H10 for RANKL; PeproTech, Rocky Hill, NJ, Cat# 500-P149Bt for OPG) followed by washing and incubation with streptavidin peroxidase and a fluorogenic substrate solution (Thermo Scientific, Waltham, MA, Cat# 15,169). Free sRANKL and OPG were quantified by interpolation from standard curves (using Amgen recombinant huRANKL or Cat# 185-OS/CF from R&D) for each plate. The lower limit of quantitation for sRANKL assay was 1.5 pg/ml. Similar procedures were applied for detection of sRANKL/OPG complex captured with anti-huRANKL mAb 74F1 (Amgen Inc.).

## 4 Outcomes

### 4.1 Statistical Analysis

Differences between *BRCA*-mutation carriers and wild-type controls as a function of the menstrual cycle were assessed using linear regression models for each hormone and allowing hormone levels to vary differentially over time for cases and controls. Specifically, the log hormone levels were regressed on restricted cubic spline functions of time using three internal knots, interacted with case–control status and age. The knots were placed where a local polynomial model suggested P turning points (days 7 and 22) and where the luteal phase typically begins (day 13), although spline functions are largely insensitive to knot placement (Lambert and Royston, 2009). Likelihood ratio tests (LRTs) compared the full model with a nested model allowing no case–control interaction with time functions and to dynamically test the null hypothesis of no difference between *BRCA*-mutation carriers and controls. There were five degrees of freedom for the LRTs; the full model contained an additional four interaction terms with the four spline functions and one individual case–control factor term. Analyses were repeated using trigonometric functions (sine and cosine) of time, as the spline approach does not guarantee endpoint convergence, although trigonometric functions may not capture dynamic trends sufficiently. LRTs here had three degrees of freedom.

To visualise the spline (or trigonometric) functions, fitted mean curves for each mutational status type, fixed at the mean age of 42.2, were plotted and superimposed upon individual data points separately coded for *BRCA1*-carriers, *BRCA2*-carriers, and controls. Further, a mean difference curve (mean[log(value_*BRCA*_)] − mean[log(value_*control*_)]) was plotted for each hormone, comparing each group of interest. For both plot types, 95% confidence bands assumed normality of mean response and were calculated with Stata's *margin* command, using a factor or contrast operator, respectively, set to the necessary values of spline functions and age.

By summing the absolute deviations of the difference curves over the menstrual cycle, we estimated differential expression between cases and controls over vectors of the relevant hormones. Because all hormones were logged, difference estimates were based on a common scale, the log(ratio difference). This sum of absolute differences was estimated by fitting a multivariate regression model and using an LRT on 20° of freedom to test the overall difference between cases and controls over the hormone vector. The vector of absolute differences follows a multivariate *folded*-normal distribution from which correct inferences can be difficult to draw. We therefore approximated the sum and associated 95% confidence band with 1000 bootstrap samples, using the 50th, 2.5th, and 97.5th centiles, respectively. The bootstrap median (or mean) was slightly larger than the simple sum of absolute differences calculated from each individual hormone because the absolute mean of a normal distribution is always less than that of its corresponding folded-normal distribution. Similarly, the appropriate null hypothesis value (based on a multivariate half-normal distribution) is not zero, but larger, ensuring positive values for the sum of absolute deviations. We estimated this null hypothesis value for each day of the cycle by simulation from a multivariate normal distribution (n = 100.000) using a mean vector of zeroes and a covariance matrix containing the set of estimated variance-covariance terms from the multivariate model.

Tests of mean difference between each treatment group of experimental animals were performed with a Dunnett-type adjustment for multiple comparisons to a control.

We used the reported hazard ratios (HR) for breast cancer based on the nucleotide position of the *BRCA1* and *BRCA2* mutation (Rebbeck et al., 2015). The estimated HRs indicating the risk for breast cancer at specific regions of the *BRCA1*/*2* mutation was regressed on the log of free serum OPG (pg/ml), adjusted for age at sample at cycle day. The use of fractional polynomials indicated that a linear fit was appropriate.

## 5 Role of the Funding Source

The work was in part sponsored by Amgen Inc. Amgen Inc. employees (YP, PY, and WCD) were involved in the study design, data collection, and data analysis (ie RANKL and OPG assays in human and animal tissues), data interpretation, and writing of the report. The corresponding author had full access to all the data in the study and had final responsibility for the decision to submit for publication. He is accountable for all aspects of the work in ensuring that questions related to the accuracy or integrity of any part of the work are appropriately investigated and resolved.

## 6 Results

**Fig. 1:**
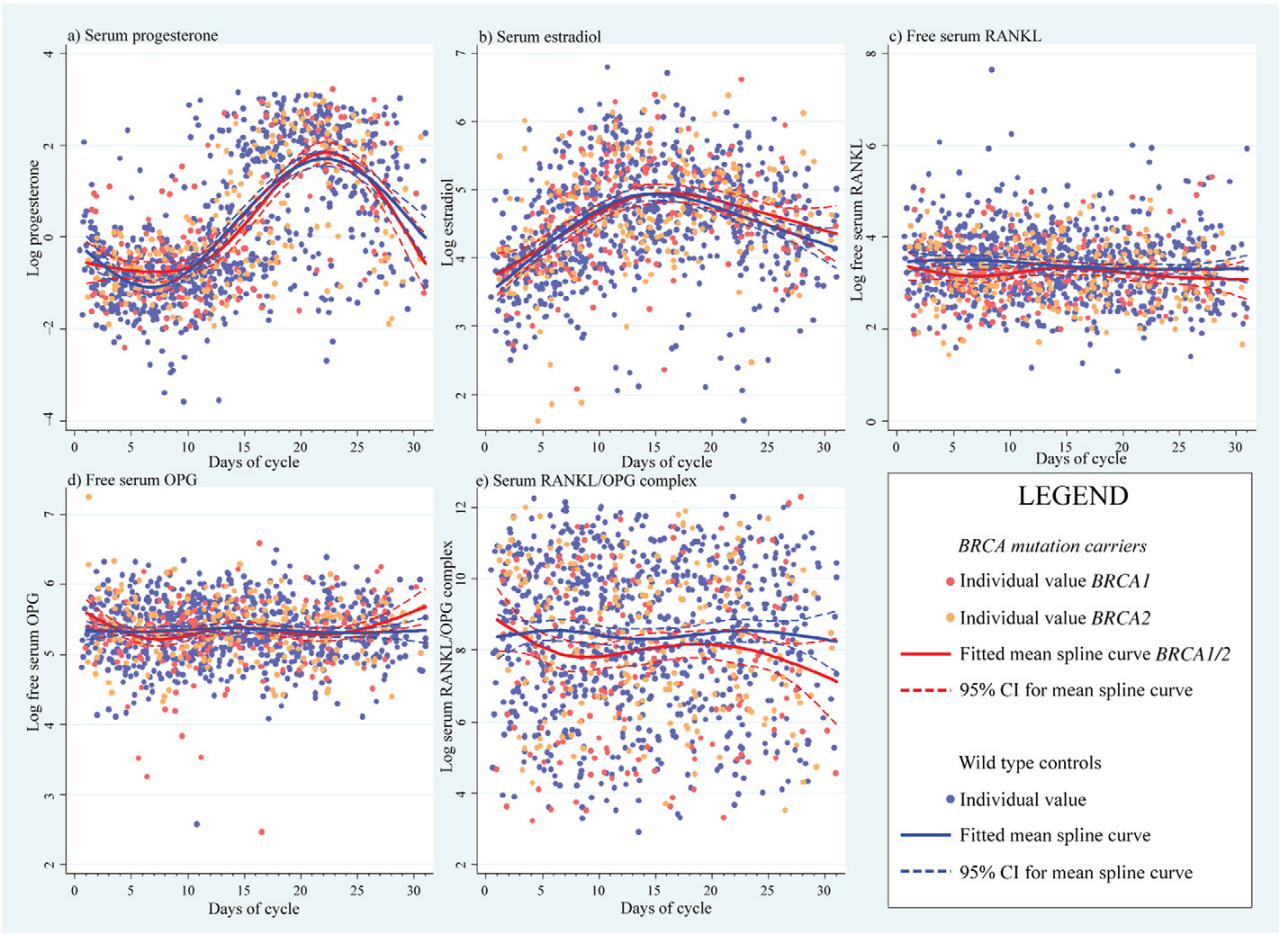
Serum analysis of hormones. Analysis of (log) (a) progesterone, (b) oestradiol, (c)RANKL, (d) OPG, and (e) RANKL/OPG complex in serum from women with and without a mutation in the *BRCA1* or *BRCA2* gene as a function of the menstrual cycle. The ranges of concentrations were: sRANKL (2.9 pg/ml–2135 pg/ml), sOPG (10.8 pg/ml–1414 pg/ml) and sRANKL/OPG complex (17.4 pg/ml–225,101 pg/ml). RANKL = receptor activator of NF-κB ligand, OPG = osteoprotegerin, and CI = confidence interval.

The mean log measurements of serum P, E2, free RANKL, free OPG, and RANKL/OPG complex were fitted as a function of time, with separate mean curves for *BRCA*-mutation carriers vs controls. Overlays of curves illustrating individual data points of mutation carriers, and controls showed typical menstrual cycle dynamics for P and E2, but free RANKL, free OPG and RANKL/OPG complex did not vary substantially throughout the menstrual cycle (Fig. 1).

**Supplemental Fig. 1:**
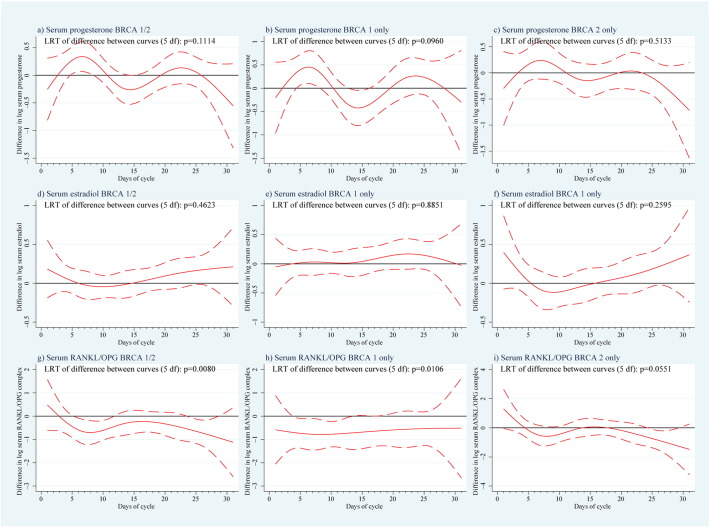
Case control differences in hormone values. Mean difference in (log) progesterone, oestradiol and RANKL/OPG complex in serum between women with and without a mutation in the *BRCA1* and/or *BRCA2* gene as a function of the menstrual cycle, with 95% confidence bands. LRT = likelihood ratio test, df = degrees of freedom, RANKL = receptor activator of NF-κB ligand, and OPG = osteoprotegerin.

**Fig. 2:**
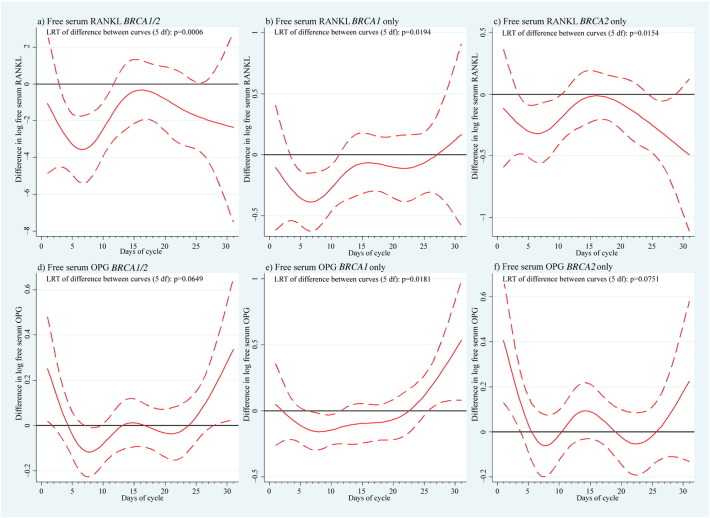
Case–control differences in hormone values. Mean differences in (log) (a–c) RANKL and (d–f) OPG in serum between women with and without a mutation in the *BRCA1* and/or *BRCA2* gene as a function of the menstrual cycle, with 95% confidence bands. RANKL = receptor activator of NF-κB ligand, LRT = likelihood ratio test, df = degrees of freedom, and OPG = osteoprotegerin.

We examined potential hormone level variations between *BRCA*-mutation carriers and controls from difference curves over the menstrual cycle with 95% confidence bands (Fig. 2; Supplemental Fig. 1). Overall, the LRT for P was not significantly different between cases and controls (p = 0.1114, Supplemental Table 1), although the difference plots showed significantly higher levels in *BRCA*-mutation carriers around days 6 and 24 (especially *BRCA1*). The LRT for E2 showed no difference between cases and controls (p = 0.4623); however, levels tended to be higher in the follicular phase, mostly for *BRCA2*. All mutation carriers had lower mean values of free serum RANKL than controls (p = 0.0006), particularly in the early- to mid-follicular phase. Overall difference in the mean curves (p = 0.0649) for free serum OPG was indicated, while OPG levels were significantly lower throughout the menstrual cycle in *BRCA1*-mutation carriers. Serum RANKL/OPG complex values were consistently lower in *BRCA* carriers (p = 0.008) throughout the cycle. This was the only variable for which the spline function-fitted mean curves did not display endpoint-convergence. Overall conclusions based on LRT p-values (Supplemental Table 1) and mean curve shapes were broadly similar using trigonometric functions. Stratification by *BRCA1*/*2* status revealed no significant difference, although differences vs controls appeared greater for *BRCA1*-mutation carriers, most notably for P and OPG.

**Supplemental Fig. 2:**
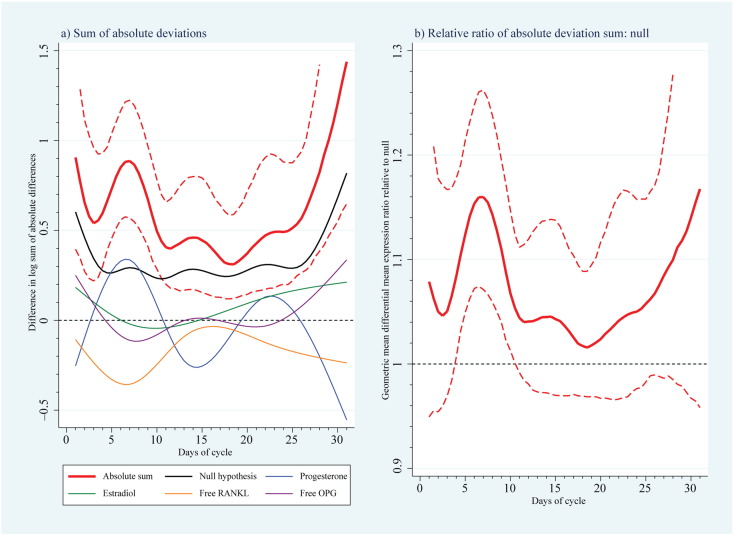
Sum of absolute differences in hormone values. a) Sum of absolute mean differences in log serum progesterone, oestradiol, OPG, and RANKL between women with and without a mutation in the *BRCA1* and/or *BRCA2* gene as a function of the menstrual cycle with bootstrapped 95% confidence bands, and individual component serum differences. Black line represents the appropriate null value for comparison, based on a multivariate half-normal distribution. b) The ratio of the (geometric) mean differential expression relative to the null. RANKL = receptor activator of NF-κB ligand and OPG = osteoprotegerin.

**Supplemental Fig. 3:**
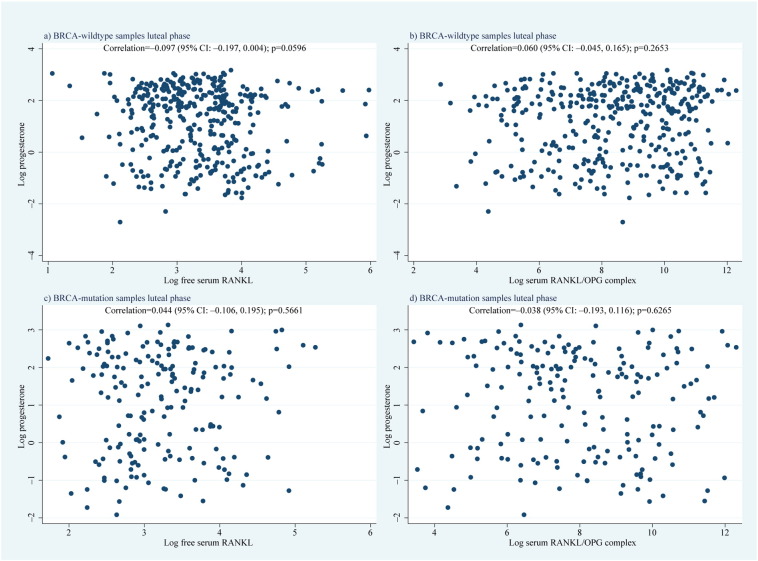
Correlation of serum progesterone and RANKL (a,c) or RANKL/OPG complex (b,d). Correlation between luteal phase serum progesterone and free serum RANKL or RANKL/OPG complex in human (a,b) BRCA wildtype and (c,d) *BRCA1/2* mutation carriers. CI = confidence interval, RANKL = receptor activator of NF-κB ligand, and OPG = osteoprotegerin.

**Supplemental Fig. 4:**
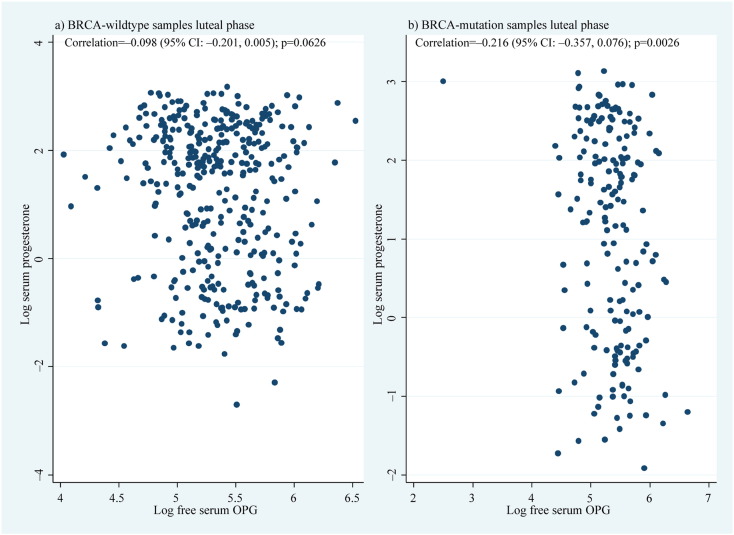
Correlation of serum progesterone and OPG. Correlation between luteal phase serum progesterone and free serum OPG in human (a) *BRCA* wild-type and (b) *BRCA1/2* mutation carriers. CI = confidence interval and OPG = osteoprotegerin.

Supplemental Fig. 2a depicts the sum of absolute differences for all serum hormones analysed, with an approximate null hypothesis curve for inferential comparison. There was strong evidence of differential levels across the four distinct hormones analysed between *BRCA1*/*2*-mutation carriers and controls (multivariate LRT p = 0.0007), most noticeable during days 4–11, with the peak at 7 days corresponding to a 16.0% (95% CI: 7.1%–26.2%) geometric mean difference in hormone expression vs the null (Supplemental Fig. 2b). No serum RANKL peak was apparent in the luteal phase (Fig. 1), but a clear inverse pattern of different levels between mutation carriers and controls regarding OPG and P was observed (Fig. 2d–f; Supplemental Fig. 1a–c). Correlation of serum P with serum RANKL, OPG, and RANKL/OPG in the luteal phase separately for controls and mutation carriers showed no association between serum P and RANKL or RANKL/OPG (Supplemental Fig. 3). Serum P and serum OPG demonstrated a negative association that was much more significant in *BRCA*-mutation carriers (*rho* = − 0.216; p = 0.002) vs controls (*rho* = − 0.098; p = 0.06; Supplemental Fig. 4).

Supplemental Fig. 2a depicts the sum of absolute differences for all serum hormones analysed, with an approximate null hypothesis curve for inferential comparison. There was strong evidence of differential levels across the four distinct hormones analysed between *BRCA1*/*2*-mutation carriers and controls (multivariate LRT p = 0.0007), most noticeable during days 4–11, with the peak at 7 days corresponding to a 16.0% (95% CI: 7.1%–26.2%) geometric mean difference in hormone expression vs the null (Supplemental Fig. 2b). No serum RANKL peak was apparent in the luteal phase (Fig. 1), but a clear inverse pattern of different levels between mutation carriers and controls regarding OPG and P was observed (Fig. 2d–f; Supplemental Fig. 1a–c). Correlation of serum P with serum RANKL, OPG, and RANKL/OPG in the luteal phase separately for controls and mutation carriers showed no association between serum P and RANKL or RANKL/OPG (Supplemental Fig. 3). Serum P and serum OPG demonstrated a negative association that was much more significant in *BRCA*-mutation carriers (*rho* = −0.216; p = 0.002) vs controls (*rho* = − 0.098; p = 0.06; Supplemental Fig. 4).

**Fig. 3:**
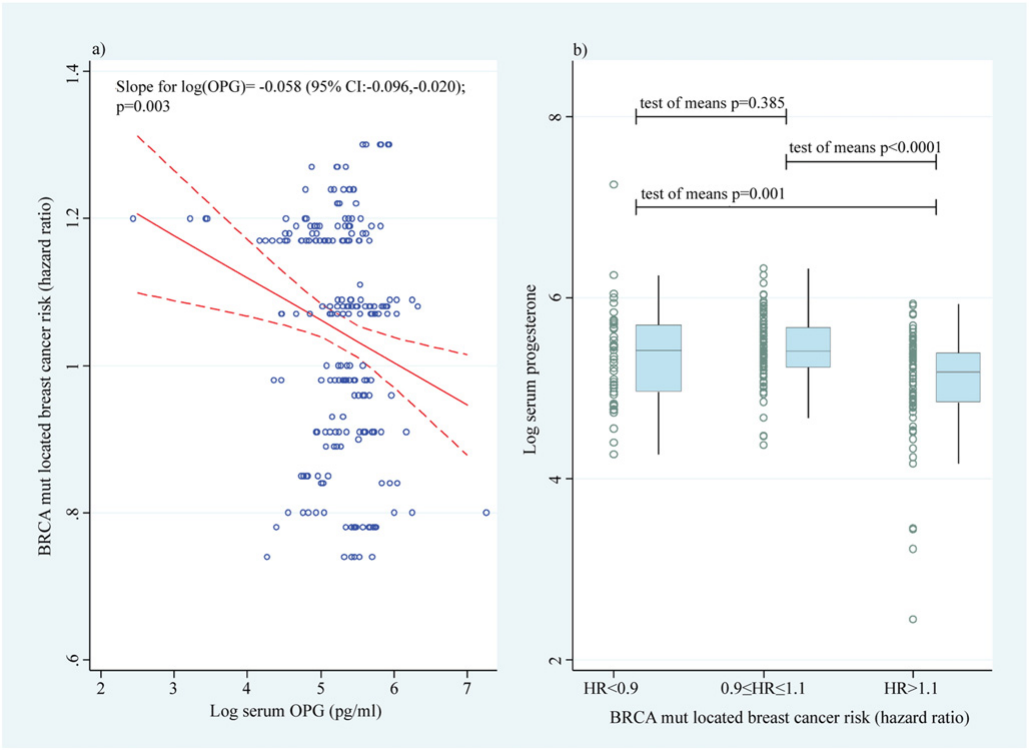
Association between the nucleotide position of the *BRCA1*/*2* germline mutation and serum OPG levels. The estimated hazard ratio (HR) indicating the risk for breast cancer depending on the nucleotide position of the *BRCA1*/*2* mutation was regressed on the log of free serum OPG (pg/ml), adjusted for age and menstrual cycle day at sample donation (a). Serum samples from human volunteers have been split according to the HR (< 0.9; < 0.9–1.1; > 1.1) given by the volunteers nucleotide position of the *BRCA* mutation and serum OPG levels blotted for each group. OPG = osteoprotegerin.

Recent evidence indicates that *BRCA1*/*2* mutation triggered breast cancer risk also depends on the actual site of the germline mutation (Rebbeck et al., 2015). In order to test whether the breast cancer risk within the *BRCA*-mutation carriers might be associated with serum OPG levels we performed a linear regression analysis (adjusted for age and menstrual cycle day) of serum OPG levels and reported breast cancer risk associated with specific sites of mutations in the 222 samples for which we had exact information on the site of the mutation (Rebbeck et al., 2015). We observed a significant decrease in reported HR for breast cancer risk with increasing serum OPG levels (beta = − 0.058; 95% CI: − 0.020, − 0.096; p = 0.003; Fig. 3).

**Fig. 4:**
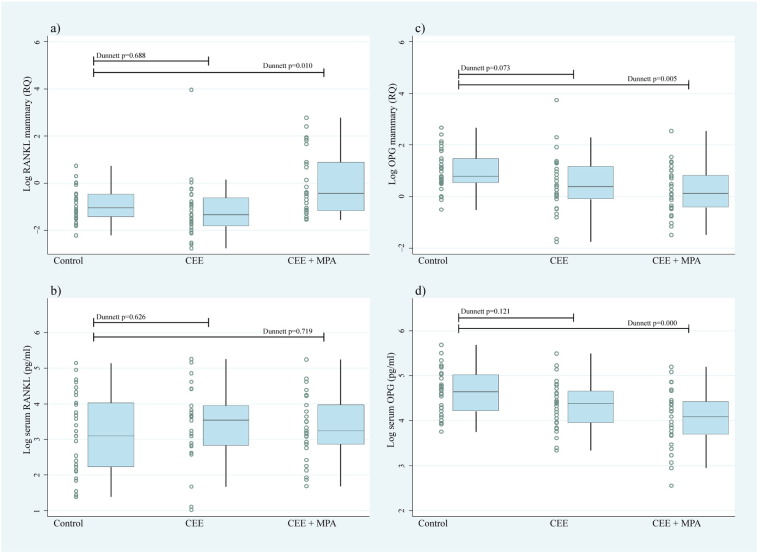
Hormonal effects on RANKL and OPG in tissue and serum. Expression of mammary gland tissue (a) *RANKL* and (c) *OPG* mRNA and matched serum (b) RANKL and (d) OPG concentrations in ovariectomised adult female cynomolgus macaques treated for two years with placebo (control; n = 31), CEE at 0.042 mg/kg (n = 28) or CEE + MPA at 0.167 mg/kg (CEE + MPA; n = 29). Tests of group mean differences adjusted by Dunnett method for multiple comparisons with a control. The ranges of concentrations were: sRANKL (2.8 pg/ml − 191 pg/ml), sOPG 12.8 pg/ml-293 pg/ml). RANKL = receptor activator of NF-κB ligand, OPG = osteoprotegerin, CEE = conjugated equine oestrogens, and MPA = medroxyprogesterone acetate.

Because P was negatively associated with serum OPG, especially in *BRCA*-mutation carriers, but the well-established P-mediated increase in RANKL in breast tissue was not mirrored in serum, we examined serum and mammary gland tissues from ovariectomised adult female cynomolgus macaques treated with no hormones (control), oestrogen only (CEE) or oestrogen/progestin (CEE + MPA) for 2 years. Whereas progestin treatment led to higher RANKL expression in the mammary gland (Fig. 4a), no differences in serum RANKL were observed between the three groups (Fig. 4b). In contrast, OPG was significantly lower post-progestin treatment in both breast (Fig. 4c) and serum (Fig. 4d).

**Supplemental Fig. 5:**
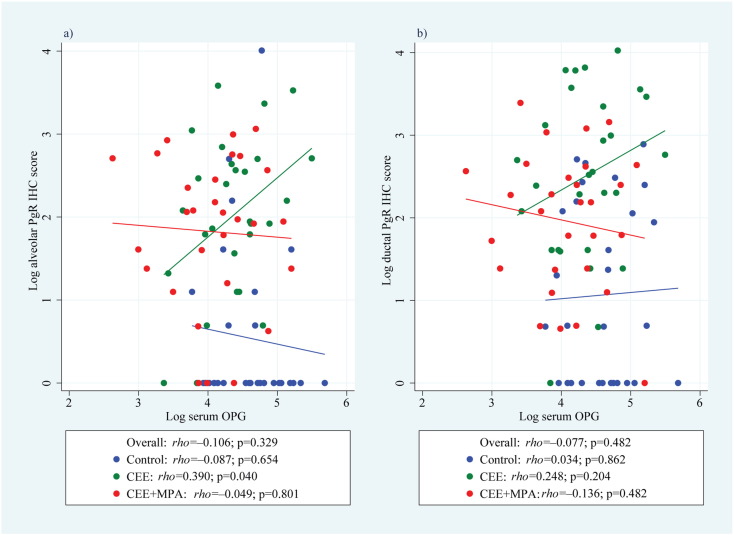
Correlation of mammary gland progesterone receptor (PgR) expression in (a) alveolar and (b) ductal cells in cynomolgus macaques. PgR = progesterone receptor, IHC = immunohistochemical, OPG = osteoprotegerin, CEE = conjugated equine oestrogens, and MPA = medroxyprogesterone acetate

**Fig. 5:**
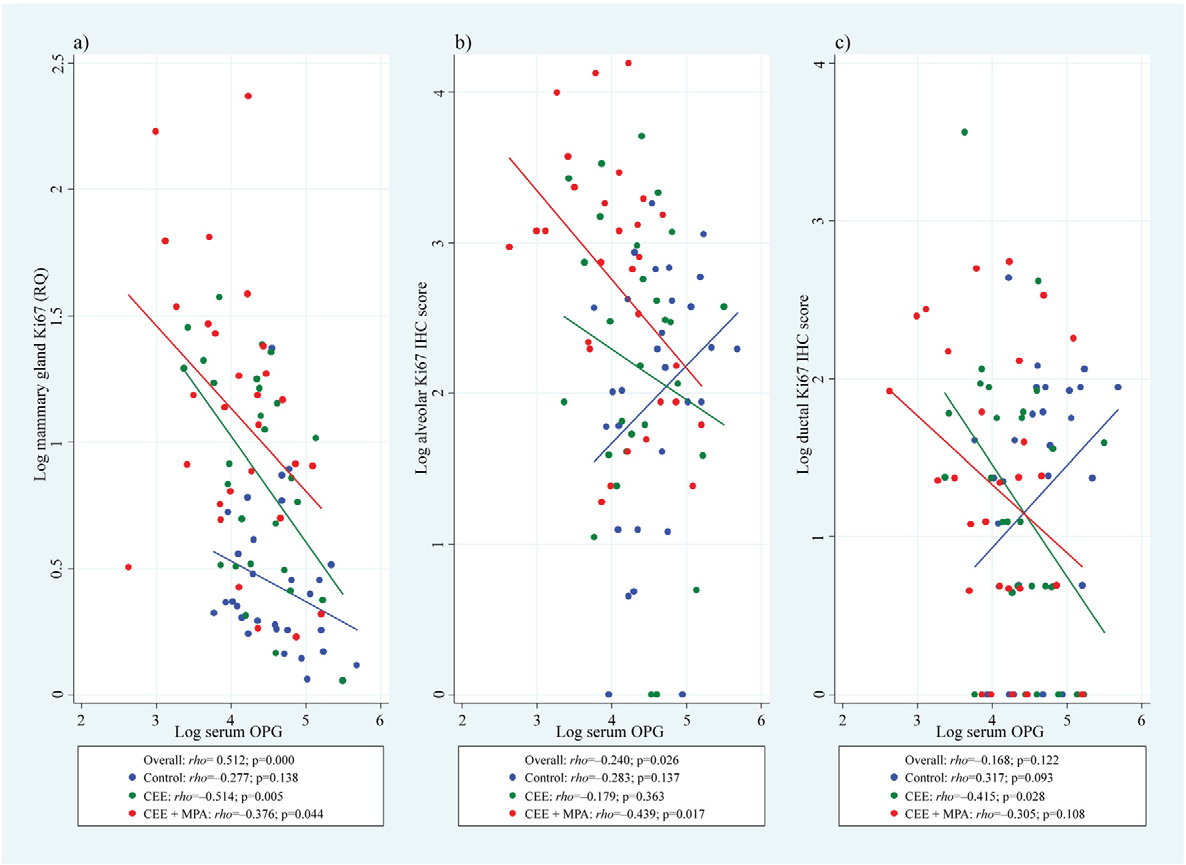
Serum OPG and mammary gland proliferation. Correlation between serum OPG and mammary gland Ki67 (a) mRNA and IHC labelling in (b) alveolar epithelial cells and (c) ductal epithelial cells in ovariectomised adult female cynomolgus macaques treated for two years with placebo (control), CEE at 0.042 mg/kg CEE or CEE + MPA at 0.167 mg/kg (CEE + MPA). IHC = immunohistochemical, OPG = osteoprotegerin, CEE = conjugated equine oestrogens, and MPA = medroxyprogesterone acetate.

We then evaluated whether serum OPG was associated with increased mammary epithelial proliferation, and found a significant inverse correlation (*rho* = − 0.545, p < 0.001; Fig. 5a) between serum OPG and mammary gland Ki67 mRNA expression. Although we cannot distinguish the effects of OPG from direct effects of P on proliferation, the significant inverse correlations between serum OPG and mammary gland proliferation with either oestrogen alone or oestrogen/progestin (Fig. 5a), plus the significant inverse correlations between serum OPG and Ki67 expression within both alveolar (Fig. 5b) and ductal (Fig. 5c) epithelia in both treatment groups (p < 0.05 for both), suggest an effect of OPG independent of PgR signalling (Supplemental Fig. 5).

**Fig. 6:**
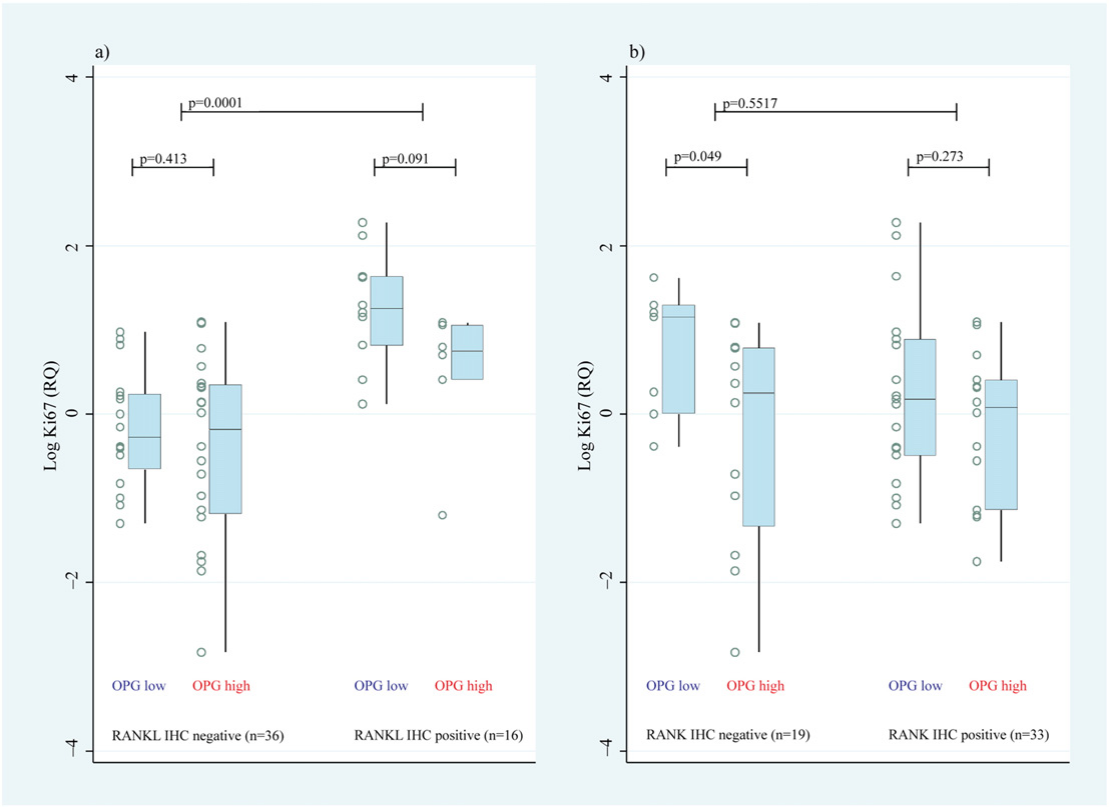
Mammary gland proliferation stratified by RANKL and RANK expression in the mammary gland and OPG levels in the serum. Cynomolgus macaques were divided according to expression of (a) RANKL and (b) RANK in their mammary gland and then substratified according to the serum OPG level (high/low, above and below the median). OPG = osteoprotegerin, RANKL = receptor activator of NF-κB ligand, IHC = immunohistochemical, and RANK = receptor activator of NF-κB.

A strong association was noted between mammary tissue RANKL and Ki67 expression (Fig. 6a), but not with RANK (Fig. 6b). In animals expressing RANKL in the mammary gland, low (< median) serum OPG levels trended (p = 0.09) towards higher mammary gland proliferation (Fig. 6a).

**Fig. 7:**
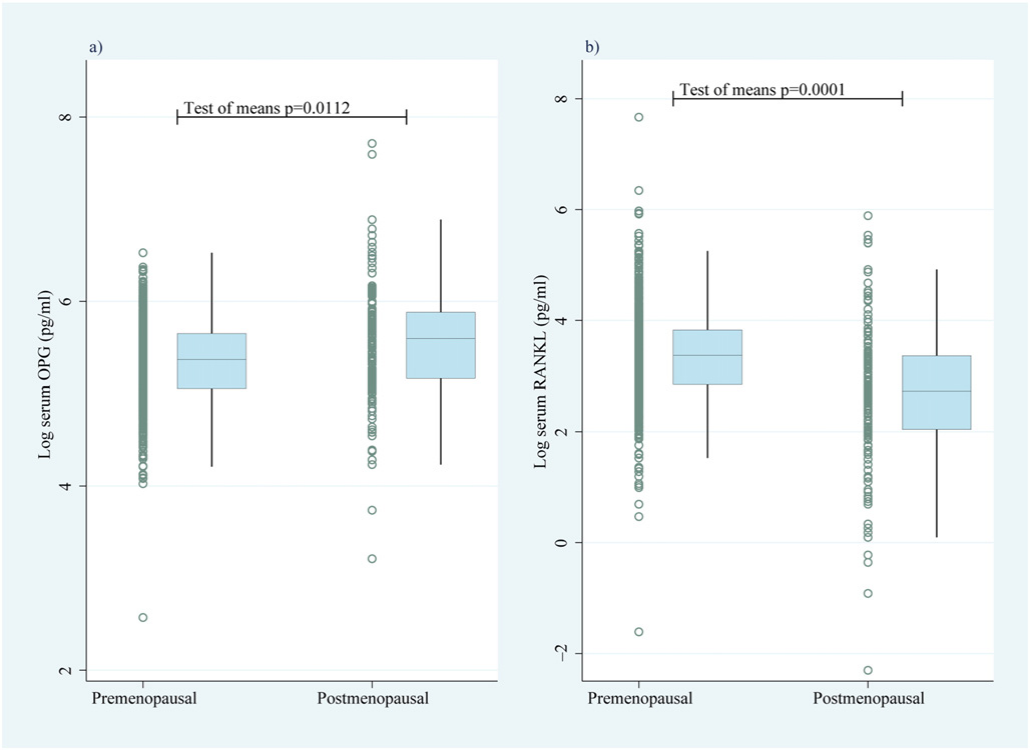
Serum levels of OPG and RANKL. Serum levels of (a) OPG and (b) RANKL in premenopausal (n = 832) and postmenopausal (n = 150) women. The ranges of concentrations in postmenopausal women were: sRANKL (2 pg/ml–361 pg/ml) and sOPG (42 pg/ml–2237 pg/ml). OPG = osteoprotegerin and RANKL = receptor activator of NF-κB ligand.

Finally, evaluation of OPG levels in postmenopause (a state known to protect from breast cancer) suggested that serum OPG levels are significantly (p = 0.01) higher (Fig. 7a) in postmenopause vs premenopause; the opposite tendency (p = 0.0001) was true for serum RANKL (Fig. 7b).

## 7 Discussion

Hormonal factors, particularly exposure to P, may influence breast cancer risk associated with *BRCA* mutations (Poole et al., 2006, Widschwendter et al., 2013). We found that dysregulation of OPG may mediate this relationship. We examined serum RANKL and OPG levels across the menstrual cycle and their associations with hormonal responsiveness in the mammary gland. OPG was dysregulated in *BRCA*-mutation carriers and inversely associated with breast cancer risk and mammary epithelial proliferation, as suggested by the aberrantly low OPG through most of the menstrual cycle in mutation carriers (in particular in those at extremely high risk for breast cancer), an inverse correlation between serum OPG and luteal-phase P levels that was more marked in *BRCA*-mutation carriers, low serum OPG levels in the animal model associated with increased mammary epithelial cell proliferation, and significantly higher OPG levels in the absence of functional ovaries (ie postmenopause). Interestingly, OPG levels in breast and serum were both decreased in presence of P. However, serum levels of RANKL do not appear to reflect local increases at the tissue level, including breast (this study) and bone (Eghbali-Fatourechi et al., 2003). These data suggest that the net magnitude of RANK signalling in the breast upon P exposure may be regulated by local increases of RANKL along with decreases in local and systemic (serum) OPG thereby increasing the RANKL:OPG ratio in this tissue (Wood et al., 2013).

RANK pathway activation by P-mediated RANKL upregulation plays an important role in breast carcinogenesis (Gonzalez-Suarez et al., 2010, Schramek et al., 2010), in part by increasing mammary stem cell proliferation (Schramek et al., 2010, Joshi et al., 2010). Moreover, deleting RANK from the mammary epithelium decreases incidence and delays onset of PgR-mediated mammary cancer (Schramek et al., 2011), indicating that RANK signalling suppression might be an excellent strategy for breast cancer prevention. Whereas anti-P treatment inhibits mammary tumourigenesis in *BRCA1*/*p53*-deficient mice by decreasing ductal branching and alveolar proliferation (Poole et al., 2006), long-term treatment of premenopausal women with selective PgR modulators has not been tested sufficiently and would probably lead to substantial side-effects, including adverse effects on the endometrium (Benagiano et al., 2014). Because OPG was not regulated throughout the menstrual cycle and luteal-phase P showed a much stronger inverse association in *BRCA*-mutation carriers vs controls, cell-autonomous factors might be involved in P-OPG regulation. Hence, our study provides strong support for the direct targeting of RANKL (ie replacing low OPG levels in mutation carriers), an idea so far only investigated in mice because of the influence of the menstrual cycle on RANKL signalling in premenopausal humans (Haynes et al., 2014, Hu et al., 2014).

Recent evidence suggests OPG deficiency may promote breast cancer development. Serum OPG analysis in > 6000 healthy subjects (Vik et al., 2015) showed an inverse relation in younger women (< 60 years) between serum OPG and risk of incident cancer at all sites, but primarily in the breast and reproductive tract (Vik et al., 2015). High OPG levels protected against breast cancer and mortality (Vik et al., 2015). An independent analysis of ~ 4500 primary breast cancers showed no prognostic value for RANK/RANKL expression, but a substantially better prognosis in oestrogen receptor-positive breast cancer cases showing high OPG expression (Sanger et al., 2014). A “suppressed” RANK pathway was likewise associated with better prognosis in another large breast cancer set (Santini et al., 2011).

Denosumab, an antibody against RANKL that mimics the physiological role of OPG, is currently in clinical use for osteoporosis and for the treatment of skeletal related events in patients with bone metastasis resulting from solid tumours. In patients with breast cancer, denosumab has recently been shown to reduce the risk of clinical fractures in postmenopausal women with breast cancer receiving aromatase inhibitors and is currently being studied for any effect on prolonging bone metastasis free survival in women with early-stage breast cancer who are at high risk for disease recurrence (Gnant et al., 2015). While our previous data highlighted the potential for antiprogestogens for breast cancer prevention, the current data additionally support a clinical breast cancer prevention trial using denosumab to compensate for the low OPG levels throughout the menstrual cycle in *BRCA*-mutation carriers.

## Contributors

MW had the idea, did the literature search, designed the study, was involved in data collection and data interpretation, wrote the first draft of the manuscript and together with MB did the statistical analysis and prepared the Figures. WCD made substantial contributions to the conception and design of the work. LF, ANR, SP, DR, DGE, UM, and IJJ collected, analysed and interpreted the human data. LD interpreted the data. MC, CEW, and WCD contributed the animal data. YP, PY, and WCD developed the serum RANKL, OPG, and RANKL/OPG assays and analysed the human and animal serum samples. All authors were involved in revising the manuscript for important critical content. All authors approved the final version of this manuscript.

## Declaration of Interests

MW, MB, LF, SP, DR, LD, MC, DGE, IJJ and CEW have no conflict of interests to declare.

ANR received honoraria from Roche, provided expert testimony for AstraZeneca, and consulted for Myriad and Abcodia.

UM has stocks and received research funding from Abcodia Ltd. which has an interest in cancer screening and biomarkers.

YP, PY, and WCD are employed by and own Amgen Inc. stocks.

The following are the supplementary data related to this article.

**Supplemental Table 1:**
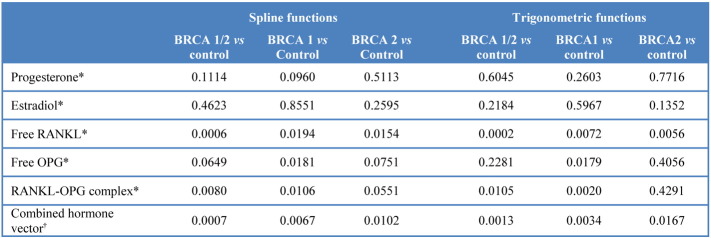
Summary of Tests of Difference between groups for mean functions of time. Likelihood ratio tests used to test between the models that include interactions of time functions and group plus individual group term and time functions versus models that only include the time functions. *LRT test of difference between groups based on 5 degrees of freedom (df) for spline functions or 3 df for trigonometric functions; ^†^LRT test of difference between groups from a multivariate model with {progesterone, estradiol, RANKL, OPG} based on 20 df for spline functions and 12 df for trigonometric functions.

Supplementary data to this article can be found online at http://dx.doi.org/10.1016/j.ebiom.2015.08.037.
